# HLA-C*06:02-independent, gender-related association of *PSORS1C3* and *PSORS1C1*/*CDSN* single-nucleotide polymorphisms with risk and severity of psoriasis

**DOI:** 10.1007/s00438-018-1435-4

**Published:** 2018-03-27

**Authors:** Andrzej Wiśniewski, Łukasz Matusiak, Aneta Szczerkowska-Dobosz, Izabela Nowak, Piotr Kuśnierczyk

**Affiliations:** 1 Laboratory of Immunogenetics and Tissue Immunology, Ludwik Hirszfeld Institute of Immunology and Experimental Therapy, Polish Academy of Sciences, Wrocław, Poland; 2 Department of Dermatology, Venereology and Allergology, Wroclaw Medical University, Wrocław, Poland; 3 Department of Dermatology, Venereology and Allergology, Medical University of Gdańsk, Gdańsk, Poland

**Keywords:** PSORS1C3, PSORS1C1/CDSN, LOC105375015, HLA-C*06:02, Single-nucleotide polymorphisms, Psoriasis vulgaris

## Abstract

Psoriasis vulgaris (PsV) is a common, chronic skin disease with a complex genetic and environmental etiology. We investigated, in 461 psoriatic patients and 454 healthy controls, the associations with psoriasis of four single-nucleotide polymorphisms (SNPs) from the psoriasis susceptibility 1 (*PSORS1*) interval: rs1062470 (*PSORS1C1*/*CDSN*), rs887466 (*PSORS1C3*), rs2894207 and rs10484554 (*LOC105375015*). The minor alleles of three SNPs (rs1062470A, rs2894207C and rs10484554T) strongly increased the disease risk (OR = 2.17, *p* < 0.0001; OR = 2.33, *p* < 0.0001 and OR = 2.68, *p* < 0.0001, respectively), whereas the minor A allele of rs887466 exerted a protective effect (OR = 0.73, *p* = 0.001). The strength of association for SNPs was the highest in patients with very early onset psoriasis (≤ 20 years), while in late onset psoriasis (> 40 years) the association was the weakest. The haplotype rs1062470A/rs887466G/rs2894207C/rs10484554T highly significantly increased the disease risk (OR = 3.58, *p* = 8.0e−027), while the haplotypes rs1062470G/rs887466A/rs2894207T/rs10484554C and rs1062470G/rs887466G/rs2894207T/rs10484554C were strongly protective (OR = 0.65, *p* = 0.002 and OR = 0.55, *p* = 2.4e−009, respectively). Additionally, we showed a HLA-C*06:02-independent gender-related effect of the rs887466A allele which was protective against psoriasis in males (OR = 0.61, *p* = 9.2e−005), but not in females (*p* = 0.66). We also demonstrated a correlation of PASI score value with rs1062470 genotype, and again only in male patients (*p* = 0.006) and HLA-C*06:02-independent. Our results show, for the first time, the male-only associations of the *PSORS1C3* gene with psoriasis risk and of the *PSORS1C1*/*CDSN* gene with severity of disease. However, the age dependent associations need to be validated in larger sample sizes as well as in other populations.

**Electronic supplementary material:**

The online version of this article (10.1007/s00438-018-1435-4) contains supplementary material, which is available to authorized users.

## Introduction

**Fig. 1:**
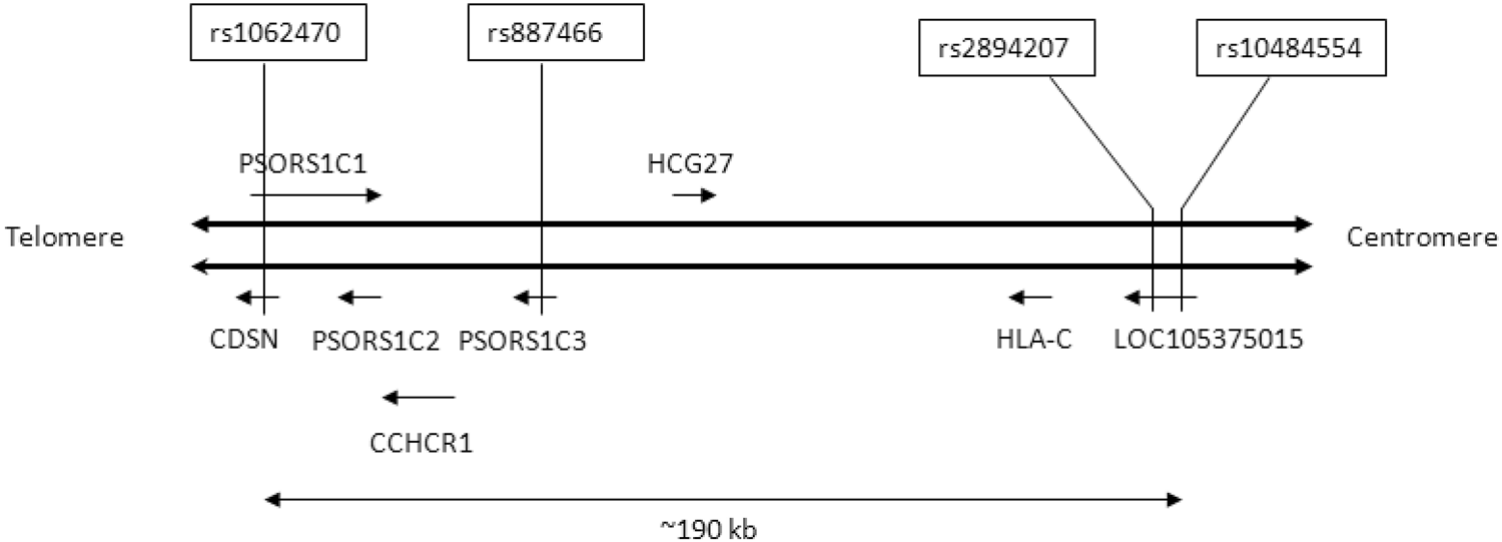
Localization of studied SNPs within *PSORS1* region on human chromosome 6

Psoriasis is a common skin disorder of multifactorial origin with prevalence in Europe between 0.73–2.9% (Veal et al. 2002; Di Meglio et al. 2014). The main genetic determinant for psoriasis, known as psoriasis susceptibility 1 (*PSORS1*), is located within the major histocompatibility complex (MHC) on chromosome 6p21.3 (Trembath et al. 1997; Nair et al. 2000), and spanning, according to different authors, from 180 to 250 kb (Nair et al. 2006; Clop et al. 2013). Although the results of genome-wide association scans (GWAS) and high-density single-nucleotide polymorphism (SNP) data repeatedly point to HLA-C*06:02 as the most likely *PSORS1* gene (Veal et al. 2002; Łuszczek et al. 2003; Nair et al. 2006; Feng et al. 2009; Capon 2017), the mechanism by which this allele predisposes to psoriasis is still poorly understood. Moreover, no HLA-C*06:02-specific antigen or interacting protein has been identified, although some candidates were recently proposed (Di Meglio et al. 2014; Harden et al. 2015; Arakawa et al. 2015; Mobbs et al. 2017). Besides *HLA-C*, the *PSORS1* interval contains several other genes including protein-coding genes, non-protein coding genes and pseudogenes (NCBI dbSNP, Nov. 2017; Capon et al. 2002; Clop et al. 2013; Capon 2017). It has been found that polymorphic variants of some of them also are associated with psoriasis. In particular, associations were observed for corneodesmosin (*CDSN*) (Tazi et al. 1999; Capon et al. 2003; Allen et al. 2005; Ameen et al. 2005; Orrù et al. 2005) and coiled-coil alpha-helical rod protein 1 (*CCHCR1*) genes (Chang et al. 2005; Tervaniemi et al. 2012). However, because of the complicated and extended linkage disequilibrium (LD) pattern in the MHC region, it is not clear whether associated markers found in these genes confer risk of psoriasis dependently or independently of HLA-C*06:02 (Ameen et al. 2005; Orrù et al. 2005; Di Meglio et al. 2014). In this study we compared the distribution of alleles, genotypes and haplotypes of four non-randomly selected SNPs from the *PSORS1* locus in 461 psoriasis patients and 454 controls. These genetic variants (rs1062470, rs887466, rs2894207 and rs10484554) were typed in our earlier work to impute the HLA-C*06:02 genotypes (Wiśniewski et al. 2018), based on a method described previously by Lai and coworkers (Lai et al. 2012). We found, similarly to Lai’s group, that one particular haplotype of these SNPs (rs1062470A/rs887466G/rs2894207C/rs10484554T) precisely pointed to the HLA-C*06:02 allele in Caucasians, including Poles (Lai et al. 2012; Wiśniewski et al. 2018). The studied SNPs are located at different points of *PSORS1* and the distance between two marginal SNPs equals ~ 190-kb, spanning almost the whole *PSORS1* interval (Fig. 1). Below we present brief characteristics for each of them.

1. rs10484554 [C>T] is located in the third exon of the still uncharacterized long non-coding RNA (lncRNA) gene *LOC105375015*, approximately 34.5 kb upstream of the *HLA-C* locus (dbSNP, Nov. 2017) (Fig. 1). Some authors have described this polymorphism as belonging to the *HLA-C* gene (Strange et al. 2010; Villarreal-Martinez et al. 2016), which, however, is in conflict with the existing data from databases (dbSNP Nov. 2017; Ensembl Nov. 2017). Strong evidence of an association for rs10484554 with psoriasis was found previously in GWAS conducted for Caucasians (Strange et al. 2010), and in a case–control study in Mexicans (Villarreal-Martinez et al. 2016).
2. rs2894207 [T>C] lies in the fifth intron of the same gene and its distance to *HLA-C* is approximately 23.8 kb. There are no published data about possible association of rs2894207 with psoriasis or other diseases except for one very recent article describing an association of this variant with nasopharyngeal carcinoma in Chinese patients (Cui et al. 2016).
3. rs887466 [G>A] is located in the first intron of the non-protein coding gene *Psoriasis susceptibility 1 candidate 3* (*PSORS1C3*), approximately 93.0-kb downstream of *HLA-C*. The function of the *PSORS1C3* gene product remains elusive, but its RNA transcript was detected both in normal and psoriatic skin (Holm et al. 2005). Nothing is known about the potential function of rs887466 or its possible association with any disease.
4. rs1062470 [G>A] is mapped in the first intron of the *Psoriasis susceptibility 1 candidate 1 (PSORS1C1)* locus and simultaneously in the second exon of the *CDSN* gene (~ 152 kb downstream from *HLA-C*). Interestingly, *PSORS1C1* and *CDSN* genes are transcribed in the opposite direction and the small *CDSN* lies in the first intron of the larger *PSORS1C1*. There are no literature data about the possible role of rs1062470 in the context of regulation and/or expression of *PSORS1C1*. In contrast, in the *CDSN* gene the synonymous substitution G to A (described previously as 971C>T) (Capon et al. 2004), although it does not alter the amino acid sequence (Tyr319Tyr), decreases the affinity of *CDSN* mRNA for the poorly characterized cytoplasmic RNA binding protein, resulting in increased transcript stability (Capon et al. 2004). To date a few studies have implicated *CDSN* gene polymorphism with PsV (Tazi et al. 1999; Capon et al. 2003; Allen et al. 2005; Ameen et al. 2005; Orrù et al. 2005), however, according to our knowledge there is only one report implicating rs1062470 with this disease (Chang et al. 2006). The *CDSN* gene, based on its genomic position in close proximity to *HLA-C* and putative biological function, is still an attractive candidate for psoriasis. Corneodesmosin is thought to play a key role in corneocyte cohesion, and its proteolysis appears to be a major event in the process of skin desquamation, which is known to be altered in PsV (Jenisch et al. 1999; Jonca et al. 2011). It has been reported that the *CDSN* gene is up-regulated in psoriatic lesions (Allen et al. 2001).

According to age of onset, psoriasis was classified into two types. Type I has early onset (before 40 years) and comprises 70% of all psoriatics. This type of psoriasis displays both strong association with HLA-C*06:02 and strong family history. Type II (also termed late-onset psoriasis) develops after the age of 40 years (Henseler and Christophers 1985; Queiro et al. 2014). This type is more sporadic and rarely familial, and its association with HLA-C*06:02 is weak (Allen et al. 2005; Queiro et al. 2014; Wiśniewski et al. 2018). In this study we stratified our patients according to age at disease onset into three subtypes to check also the association of our SNPs with very early onset psoriasis (juvenile psoriasis).

## Materials and methods

### Study population

A total of 461 patients diagnosed with psoriasis vulgaris were enrolled in the study by the Department and Clinic of Dermatology, Venereology and Allergology of Wrocław Medical University (*N* = 352), and the Department of Dermatology, Venereology and Allergology of the Medical University in Gdańsk (*N* = 109). The detailed characteristics of the patients are depicted in Table 1. The diagnosis of psoriasis was made according to well-established clinical criteria, including sharply demarcated round-oval erythematous plaques with loosely adherent silvery-white scales, especially affecting the elbows, knees, lumbosacral area, intergluteal cleft, and scalp. In any doubtful cases the diagnosis was confirmed by histological examination of the skin sample. For all patients we had information about their gender, age at disease onset and age at time of blood sampling. With respect to the second parameter the patients were divided into three subgroups: (I) very early onset psoriasis (vEOP)—up to 20 years, (II) middle early onset psoriasis (mEOP)—between 21 and 40 years, and late onset psoriasis (LOP)—above 40 years. For 324 patients we had information about the Psoriasis Area and Severity Index (PASI) (Schmitt et al. 2005; Boehncke and Schön 2015). The healthy control group included 454 blood donors who had no history of psoriasis or other dermatoses. The study was approved by bioethical committees of participating medical universities in Wrocław and Gdańsk, and all participants gave signed informed consent.

**Table 1:** Characteristics of patients and controls

	Age at onset				Controls (*N* = 454)
	vEOP ≤ 20 (*N* = 152)	mEOP 21–40 (*N* = 174)	LOP > 40 (*N* = 135)	All (*N* = 461)	
Gender, women (%)	65 (42.8)	47 (27.0)	78 (57.8)	190 (41.2)	189 (41.6)
Mean age at onset	13.9	30.2	54.0	31.8	
*N* (Median for PASI score)^a^	82 (13.05)	121 (12.4)	121 (10.3)	12.05	
Women	31 (13.0)	33 (10.3)	71 (8.9)		
Men	51 (13.6)	88 (13.2)	50 (12.05)		
Mean age at the moment of blood sampling	34.4	47.9	62.1	47.6	33.1
HLA-C*06:02 carriers (%)^b^	85.5	63.8	35.5	62.7	24.4

*vEOP* very early psoriasis, *mEOP* middle early psoriasis, *LOP* late onset psoriasis

^a^Data for 324 patients only

^b^Calculated on the basis of data published in Wiśniewski et al. (2018)

### SNP genotyping

The SNPs rs10484554, rs2894207, rs887466 and rs1062470 were genotyped using the TaqMan SNP Genotyping Assay (Applied Biosystems, Foster City, USA) as described in more detail previously (Wiśniewski et al. 2018).

### Statistical analysis

All statistical tests for alleles, genotypes and haplotypes as well as Hardy–Weinberg equilibrium (HWE) and linkage disequilibrium (LD) tests were calculated using PLINK software ver. 1.07 (Purcell et al. 2007). For HWE testing the significance threshold *p* < 0.05 was adjusted. Allelic and genotypic frequencies were compared between patients (or subgroups of patients) and controls using Fisher’s exact test (two-tailed), while to compare haplotype frequencies the Chi-square statistic was applied. The odds ratio (OR) and its 95% confidence interval (95% CI) were computed as the measure of effect size. As the PASI score data did not fit the normal distribution we applied nonparametric tests comparing the medians. In this respect the Kruskal–Wallis test with Dunn’s multiple comparisons test as a post hoc test were used to demonstrate the differences in PASI score between subgroups of patients according to age at disease onset and between genotypes of rs1062470 in males and females, whereas the Mann–Whitney test was applied to test differences of PASI score between males and females (GraphPad InStat ver. 3.06). A *p* value < 0.05 was considered significant.

## Results

### Comparison of PASI score in subgroups of patients and between genders

There was no difference in median PASI score value between the subgroups vEOP and mEOP (13.05 vs. 12.40), but we noted significant differences between vEOP and LOP (13.05 vs. 10.30, *p* < 0.01) and mEOP and LOP (12.40 vs. 10.30, *p* < 0.05) (Table 1). In addition, we found a significantly higher median PASI score value for males in comparison to females in mEOP and LOP groups (13.2 vs. 10.3, *p* = 0.014 and 12.05 vs. 8.9, *p* = 0.015, respectively), although not in vEOP (13.6 vs. 13.0, *p* = 0.68), (Table 1).

### Distribution of *PSORS1* polymorphisms in psoriasis patients and healthy controls

All tested polymorphisms were in Hardy–Weinberg equilibrium in controls, but not in cases (Supplementary Table S1). The distribution of minor alleles and genotypes of SNPs differed significantly between patients and controls (Table 2). In detail, minor alleles of rs1062470, rs2894207 and rs10484554 strongly increased the risk of psoriasis (OR = 2.17, 95% CI 1.80–2.63, *p* < 0.0001; OR = 2.33, 95% CI 1.92–2.82, *p* < 0.0001 and OR = 2.68, 95% CI 2.19–3.28, *p* < 0.0001, respectively), and these results were slightly lower than those for HLA-C*06:02 obtained for the same patients and controls in our earlier study (OR = 3.58, 95% CI 2.82–4.54, *p* < 0.0001) (Wiśniewski et al. 2018). In addition, the disease risk for less frequent genotypes of tested SNPs, i.e., rs1062470AA, rs2894207CC and rs10484554TT, was much higher than for the reference homozygous genotypes (OR = 5.25, 95% CI 3.40–8.10, *p* < 0.0001; OR = 4.88, 95% CI 3.18–7.49, *p* < 0.0001 and OR = 6.82, 95% CI 4.11–11.30, *p* < 0.0001, respectively) (Table 2). The minor A allele and recessive AA genotype of rs887466 were associated with protection (OR = 0.73, 95% CI 0.60–0.88, *p* = 0.001 and OR = 0.46, 95% CI 0.31–0.70, *p* = 0.0003, respectively) (Table 2).

**Table 2:** Distribution of genotypes and minor alleles of four *PSORS1* SNPs in psoriatic patients (*N* = 461) and controls (*N* = 454)

Genotype/MA	Cases *N* (%)	Controls *N* (%)	*p*	OR (95% CI)
rs1062470				
GG	99 (21.5)	205 (45.2)	–	1.0^a^
GA	258 (56.0)	208 (45.8)	< 0.0001	2.56 (1.90–3.47)
AA	104 (22.5)	41 (9.0)	< 0.0001	5.25 (3.40–8.10)
A	466 (50.5)	290 (31.9)	< 0.0001	2.17 (1.80–2.63)
rs887466				
GG	170 (36.8)	136 (30.0)	–	1.0^a^
GA	240 (52.1)	231 (50.9)	0.2	0.83 (0.62–1.11)
AA	51 (11.1)	87 (19.1)	0.0003	0.46 (0.31–0.70)
A	342 (37.0)	405 (44.6)	0.001	0.73 (0.60–0.88)
rs2894207				
TT	100 (21.7)	231 (50.9)	–	1.0^a^
TC	268 (58.1)	179 (39.4)	< 0.0001	3.45 (2.55–4.67)
CC	93 (20.2)	44 (9.7)	< 0.0001	4.88 (3.18–7.49)
C	454 (49.2)	267 (29.4)	< 0.0001	2.33 (1.92–2.82)
rs10484554				
CC	126 (27.3)	268 (59.0)	–	1.0^a^
CT	258 (56.0)	162 (35.7)	< 0.0001	3.38 (2.53–4.52)
TT	77 (16.7)	24 (5.3)	< 0.0001	6.82 (4.11–11.30)
T	412 (44.6)	210 (23.1)	< 0.0001	2.68 (2.19–3.28)

*MA* minor allele, *OR* odds ratio, *CI* confidence interval

^a^The reference group

### Distribution of *PSORS1* polymorphisms depending on age at disease onset

Association of the polymorphisms rs1062470, rs2894207 and rs10484554 with psoriasis was the strongest in patients with age at disease onset up to 20 years (vEOP), and the weakest in late onset psoriasis (> 40 years), (Table 3). Interestingly, the minor allele of rs887466A was associated with protection, but this effect was seen only in the two younger groups of patients, i.e., vEOP (OR = 0.52, 95% CI 0.39–0.69, *p* < 0.0001) and mEOP (OR = 0.68, 95% CI 0.53–0.88, *p* < 0.0001), and not in the LOP group (OR = 1.12, 95% CI 0.85–1.47, *p* = 0.44).

**Table 3:** Distribution of genotypes and minor alleles of four *PSORS1* SNPs in psoriatic patients according to age at disease onset (subgroups of cases vs. controls, *N* = 454)

GENOTYPE/vEOP ≤ 20				mEOP 21–40			LOP > 40		
MA	*N* (%)	*p*	OR (95% CI)	*N* (%)	*p*	OR (95% CI)	*N* (%)	*p*	OR (95% CI)
rs1062470									
GG	15 (9.9)	–	1.0^a^	34 (19.5)	–	1.0^a^	50 (37.0)	–	1.0^a^
GA	92 (60.5)	< 0.0001	6.04 (3.38–10.78)	105 (60.4)	< 0.0001	3.04 (1.97–4.69)	61 (45.2)	0.39	1.20 (0.78–1.83)
AA	45 (29.6)	< 0.0001	15.0 (7.64–29.42)	35 (20.1)	< 0.0001	5.14 (2.88–9.18)	24 (17.8)	0.005	2.40 (1.32–4.33)
A	182 (59.9)	< 0.0001	3.17 (2.43–4.15)	175 (50.3)	< 0.0001	2.15 (1.67–2.77)	109 (40.4)	0.01	1.44 (1.09–1.91)
rs887466									
GG	66 (43.5)	–	1.0^a^	68 (39.1)	–	1.0^a^	36 (26.7)	–	1.0^a^
GA	82 (53.9)	0.13	0.73 (0.49–1.07)	88 (50.6)	0.17	0.76 (0.52–1.11)	70 (51.9)	0.64	1.14 (0.72–1.80)
AA	4 (2.6)	< 0.0001	0.09 (0.03–0.26)	18 (10.3)	0.003	0.41 (0.23–0.74)	29 (21.4)	0.47	1.25 (0.72–2.20)
A	90 (29.6)	< 0.0001	0.52 (0.39–0.69)	124 (35.6)	0.004	0.68 (0.53–0.88)	128 (47.4)	0.44	1.12 (0.85–1.47)
rs2894207									
TT	14 (9.2)	–	1.0^a^	37 (21.3)	–	1.0^a^	49 (36.3)	–	1.0^a^
TC	94 (61.9)	< 0.0001	8.66 (4.78–15.70)	106 (60.9)	< 0.0001	3.69 (2.42–5.63)	68 (50.4)	0.006	1.79 (1.18–2.71)
CC	44 (28.9)	< 0.0001	16.5 (8.33–32.65)	31 (17.8)	< 0.0001	4.39 (2.47–7.82)	18 (13.3)	0.05	1.92 (1.02–3.61)
C	182 (59.9)	< 0.0001	3.58 (2.73–4.69)	168 (48.3)	< 0.0001	2.24 (1.73–2.88)	104 (38.5)	0.006	1.50 (1.13–1.99)
rs10484554									
CC	17 (11.2)	–	1.0^a^	46 (26.4)	–	1.0^a^	63 (46.7)	–	1.0^a^
CT	99 (65.1)	< 0.0001	9.63 (5.55–16.70)	102 (58.7)	< 0.0001	3.66 (2.41–5.46)	57 (42.2)	0.06	1.49 (0.99–2.25)
TT	36 (23.7)	< 0.0001	23.64 (11.6–48.2)	26 (14.9)	< 0.0001	6.31 (3.33–11.93)	15 (11.1)	0.01	2.65 (1.31–5.36)
T	171 (56.3)	< 0.0001	4.27 (3.24–5.62)	154 (44.3)	< 0.0001	2.63 (2.03–3.42)	87 (32.2)	0.003	1.58 (1.17–2.12)

*MA* minor allele, *OR* odds ratio, *CI* confidence interval

^a^The reference group

### *PSORS1* haplotypes in patients and controls

In our population, we found eight *PSORS1* haplotypes with frequencies higher than 1% (Table 4). Three of them differed significantly in frequency between patients and controls. Of note, the strongest association with disease was observed for the haplotype H2 rs1062470A/rs887466G/rs2894207C/rs10484554T (OR = 3.58, 95% CI 2.79–4.55, *p* = 8.0e−027), which indicated the presence of the HLA-C*06:02 allele. The other two haplotypes (H6 and H8) were strongly protective (OR = 0.65, 95% CI 0.49–0.86, *p* = 0.002 and OR = 0.55, 95% CI 0.45–0.67, *p* = 2.4e−009, respectively) (Table 4).

**Table 4:** Estimated *PSORS1* haplotypes frequency for psoriatic patients (*N* = 461) and controls (*N* = 454)

Haplotype ID	Haplotype sequence	Cases (%)	Controls (%)	*p* value*	OR (95% CI)	Omnibus *p* value (7 *df*)
H1	GACT	10.66	10.50	0.91	–	
H2	AGCT	34.04	12.62	8.0e−027	3.56 (2.79–4.55)	
H3	AACC	24.33	28.81	0.55	–	
H4	GACC	1.52	2.07	0.37	–	1.6e−023
H5	AATC	11.33	13.37	0.18	–	
H6	GATC	11.43	16.49	0.002	0.65 (0.49–0.86)	
H7	AGTC	2.92	3.26	0.67	–	
H8	GGTC	25.66	38.80	2.4e−009	0.55 (0.45–0.67)	

*OR* odds ratio, *CI* confidence interval, *DF* degrees of freedom

*Chi-square test (*df* = 1), SNPs in haplotypes were listed in order corresponding their location on chromosome: rs1062470, rs887466, rs2894207, rs10484554; H2 haplotype (AGCT) indicates the HLA-C*06:02 allele

### Distribution of *PSORS1* polymorphisms depending on gender

There were no differences in distribution of alleles and genotypes of the tested SNPs and HLA-C*06:02 allele when our patients and controls were stratified by gender, except for rs887466 *(PSORS1C3*). The minor A allele of this SNP, which was protective against psoriasis when all patients were combined, was in fact protective only in males (OR = 0.61, 95% CI 0.47–0.78, *p* = 9.2e−005) (Table 5). In comparison to rs887466GG genotype adjusted as a baseline, the strongest effect of protection was seen for AA homozygotes (OR = 0.34, 95% CI 0.19–0.59, *p* = 0.0001) and somewhat weaker for GA heterozygotes (OR = 0.59, 95% CI 0.40–0.87, *p* = 0.007) (Supplementary Table S2).

**Table 5:** Minor allele frequency of four tested *PSORS1* markers stratified by gender

Marker	Women				Men			
	MAF Ca (%)	MAF Co (%)	*p*	OR (95% CI)	MAF Ca (%)	MAF Co (%)	*p*	OR (95% CI)
rs1062470	51.8	32.5	7.4e−008	2.23 (1.66–2.99)	49.6	31.5	1.5e−009	2.14 (1.67–2.74)
rs887466	40.5	42.0	0.66	0.93 (0.70–1.25)	34.7	46.4	9.2e−005	0.61 (0.47–0.78)
rs2894207	51.0	28.3	1.5e−010	2.64 (1.95–3.56)	47.9	30.1	2.4e−009	2.13 (1.66–2.73)
rs10484554	45.0	24.0	1.3e−009	2.58 (1.89–3.52)	44.4	22.4	2.3e−014	2.76 (2.12–3.60)
HLA-C*06:02	32.9	13.8	4.7e−010	3.07 (2.13–4.41)	35.1	11.9	4.2e−019	4.00 (2.91–5.49)

*MAF Ca* minor allele frequency for cases, *MAF Co* minor allele frequency for controls, *OR* odds ratio, *CI* confidence interval

### Correlation of rs1062470 (*PSORS1C1*/*CDSN*) genotypes with PASI score in males but not in females

**Fig. 2:**
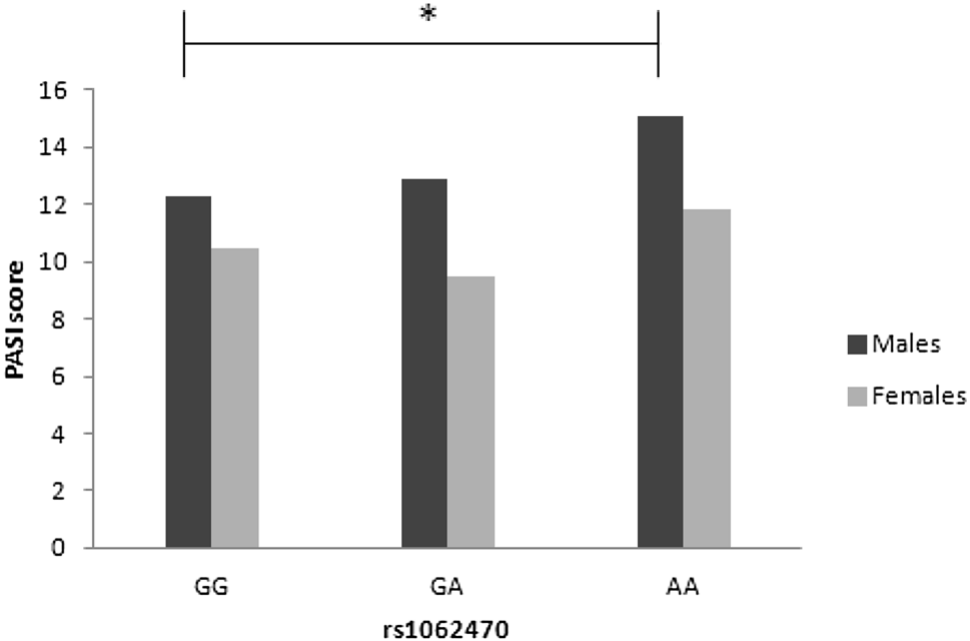
Differences in PASI score between the genotypes of rs1062470 in males and females. Data are shown as medians. Significant difference was shown as *(*p* < 0.05)

We did not observe any correlation between studied SNPs and PASI score in the group of all patients or in three subgroups with respect to age at disease onset (data not shown). However, stratification by gender demonstrated the correlation of PASI score with rs1062470 in males (*p* = 0.006) (Table 6), but not in females (*p* = 0.97) (Supplementary Table S3). In contrast, other SNPs tested here, and the HLA-C*06:02 allele as well, did not correlate with PASI score in either gender (Table 6 and S3). Additional statistics for male patients revealed significant differences in median PASI score between rs1062470 genotypes (*p* = 0.03). In detail, median values of PASI score for GG homozygotes, GA heterozygotes and AA homozygotes were 12.3, 12.9, and 15.05, respectively. A significant difference was observed between genotypes GG and AA (*p* < 0.05), while the differences between GG and GA and between GA and AA were not significant (Fig. 2).

**Table 6:** Correlation of four *PSORS1* SNPs with PASI score value in psoriatic males (*N* = 189)

SNP	BETA	SE	*R* ^2^	*T*	*P*
rs1062470	2.021	0.732	0.0391	2.759	0.006
rs887466	1.075	0.743	0.0110	1.446	0.149
rs2894207	0.306	0.736	0.0009	0.416	0.677
rs10484554	0.545	0.742	0.0028	0.734	0.463
HLA-C*06:02	0.852	0.849	0.0053	1.003	0.317

*BETA* regression coefficient, *SE* standard error, *R*^*2*^ regression r-sqared, *T* Wald test (based on t-distribution), *p* Wald test asymptotic *p* value

## Discussion

In this study we found significant associations of four SNPs located in the three genes within *PSORS1* with psoriasis risk both in single SNP and haplotype approaches. This association, like HLA-C*06:02 (our earlier report, Wiśniewski et al. 2018), was the strongest for juvenile psoriasis (≤ 20 years). The strength of association decreased in patients with age of onset 20–40 years, while in the late onset psoriasis group (> 40 years) the association was the weakest, but still significant except for rs887466. The minor A allele and AA genotype of this SNP very strongly reduced the risk of vEOP, a little less of mEOP, but not of LOP. This finding is in agreement with previous reports that polymorphisms within the *PSORS1* locus are predominantly associated with type I psoriasis but not (or weakly) with type II (Łuszczek et al. 2003; Allen et al. 2005; Chang et al. 2005, 2006; Lysell et al. 2013). Rs10484554 within *LOC105375015* was in fact the most strongly associated with the disease, although the odds ratio for the rs10484554T minor allele was lower than that for the HLA-C*06:02 allele achieved for the same patients and controls in our previous study (Wiśniewski et al. 2018). The function of this SNP is unknown, but its location in very close proximity to the exon/intron junction may suggest a role in the splicing process. Regarding intronic rs2894207, the minor C allele also increased the risk of psoriasis but less than rs10484554T. Both polymorphisms are in strong LD in our population, and the contribution of these SNPs to psoriasis observed in this study is probably due to medium-strong LD between them and HLA-C*06:02 (Supplementary Tables S4 and S5). Of note, even stronger LD (*r*^2^ = 0.7) between rs10484554 and HLA-C*06:02 was observed in the study of Strange and coworkers in other Caucasians (Strange et al. 2010). There are no published data about the possible role of *LOC105375015* in pathogenesis of psoriasis. As we mentioned earlier, this gene belongs to the family of lncRNAs. Taking into account the fact that numerous lncRNAs regulate gene expression through different mechanisms, including epigenetic regulation, transcriptional activation or repression, posttranscriptional modification of mRNA or modulation of protein activity (Adams et al. 2017), *LOC105375015* also may play some regulating role in the psoriatic process. However, explanation of this requires further studies.

The marker rs887466 from *PSORS1C3* was the only tested SNP associated with protection. Of note, we observed a protective effect only for AA genotype but not for GA, both if we compared all patients and controls and when the patients were divided according to age at disease onset. Notably, subjects (regardless of gender) with AA genotype are exclusively HLA-C*06:02-negative, whereas GA heterozygotes may be negative or positive for *06:02 (our genotyping data). Thus, the question is whether the A allele is a true marker of protection involving the *PSORS1C3* gene or rather its effect is due to absence of the HLA-C*06:02 risk allele on the same haplotype? In this second scenario, it is possible that the A allele may be linked with some other *HLA-C* allele that could deliver real protection. Indeed, many years ago we demonstrated a protective effect of the HLA-Cw*07 allele against psoriasis in the Polish population in smaller cohorts of patients and controls (Łuszczek et al. 2003). Unfortunately, funding of our project did not allow us to determine which *HLA-C* allele could potentially be linked with the rs887466A allele, and which one was preferentially present together with rs887466A in controls. The function of the relatively novel *PSORS1C3* gene is still unknown. Additionally, nothing is known about the role of intronic rs887466. Previously, several SNPs in this gene have been tested in psoriasis in Swedish and Chinese populations (Holm et al. 2005; Chang et al. 2006), but rs887466 has never been examined either in psoriasis or in any other disease. In the Swedish population an association with psoriasis was found for three exonic SNPs in *PSORS1C3*. Unfortunately, none of them was found to be in LD with rs887466. Of note, the association of those three SNPs with psoriasis was weaker than that of HLA-C*06:02 and wholly dependent on HLA-C*06:02 status (Holm et al. 2005). A much stronger (even comparable to HLA-C*06:02) association of the PSORS1C3*582A allele (rs887468, located in the 3′UTR) was observed in the Chinese population. The authors found very strong LD between this variation and the HLA-C*06:02 allele as well (Chang et al. 2006). In contrast, in our study the linkage disequilibrium between rs887466 and HLA-C*06:02 is relatively low [*r*^2^ = 0.10 in controls and *r*^2^ = 0.21 in cases (Supplementary Tables S4 and S5)], which may indicate that in our population this SNP may exert its effect independently of HLA-C*06:02, but possibly dependently on another *HLA-C* allele.

Interestingly, we observed a different association of rs887466 with psoriasis in men and women. The protective effect of the A allele and AA genotype was visible only for men. In the literature there are only a few studies investigating differences in genetic risk between both sexes with respect to psoriasis. One of them reported that HLA-Cw6-positive women might have earlier disease onset than HLA-Cw6-positive men (Gudjónsson et al. 2002). Other research on a disease related to PsV, psoriasis arthritis (PsA), revealed differences in distribution of some genetic markers from the MHC region between sexes when patients were divided according to age at disease onset (Queiro et al. 2013). Similarly, Huffmeier and coworkers, also in PsA, observed much higher frequency of PTPN22*620W allele carriers in the subgroup of male patients than in female patients (Hüffmeier et al. 2006). The fact that in our population HLA-C*06:02 was strongly associated with psoriasis both in males and females supports the *HLA-C*-independent association of rs887466 with this disease. Taking into account the above reports, it cannot be excluded that the protective effect of the rs887466A allele observed in our study only for males may not be accidental, but the reason for this phenomenon requires additional studies.

Other dissimilarities between genders were seen also when we checked for associations of tested markers with the PASI score. We found a significant correlation between rs1062470 (*PSORS1C1*/*CDSN*) and PASI score value only in males. Male patients with AA genotype had a significantly higher PASI score in comparison to male patients with GG genotype. We did not observe such a relationship in female patients. As we mentioned earlier, the exchange of guanine for adenine may lead to increased *CDSN* transcript stability (Capon et al. 2004), and likely to higher protein expression. In our study the rs1062470AA genotype increased the risk of psoriasis over fivefold and was significantly associated with higher PASI score in males. Thus, we may hypothesize that in psoriatic men with AA genotype the presence of two alleles that potentially elevate the expression of corneodesmosin in the skin may result in increased severity of psoriasis. However, it is unclear why this effect is observed only in males. Notably, other markers tested here, including HLA-C*06:02, were not correlated with PASI values in either gender. This seems to show an effect of corneodesmosin independent of HLA-C*06:02, and corresponds to the results of Orrù et al. 2005, who described a very strong, and HLA-C*06:02-independent, association of psoriasis with the *CDSN* allele in the Sardinian population. This independence in our population is also supported by relatively low LD between these two genes.

Interestingly, in our study we also observed that male patients, independently of rs1062470 genotype, had significantly higher PASI scores than female patients except for the earliest onset of disease, and this result is in agreement with previous reports that psoriasis is more severe in men than in women (Sakai et al. 2005; Hägg et al. 2017). This is the first report describing a possible sex-dependent association of rs1062470 genotype with psoriasis severity; therefore there is a need for a further larger-scale study, including also other polymorphisms in the *CDSN* gene, to confirm our findings. In addition, the comparison of corneodesmosin expression level in the psoriatic skin both in males and females and its correlation with rs1062470 genotype could be crucial to evaluate the gender-dependent effect of this SNP on disease severity.

In haplotype analysis we observed only one high-risk haplotype (AGCT) that contained all four single alleles predisposing to the disease. Of note, this haplotype was the most frequent haplotype detected in patients and indicated the presence of the HLA-C*06:02 allele. We also described two protective haplotypes. Unfortunately, we were unable to determine whether these haplotypes correspond to other *HLA-C* alleles.

In conclusion, our results demonstrated that genetic variants of three genes within the *PSORS1* locus, i.e., *CDSN, PSORS1C3* and *LOC105375015*, are significantly associated with psoriasis. This association (similarly to HLA-C*06:02) is strongly dependent on age at disease onset and concerns predominantly type I psoriasis. In addition, for the first time, we showed that association of rs887466 *(PSORS1C3)* with psoriasis risk may be different in men and women, and that the influence of rs1062470 *(PSORS1C1*/*CDSN)* polymorphism on disease severity may also be gender dependent. To the best of our knowledge, the association of rs887466 and rs2894207 with psoriasis has never been examined before. Because of the complicated and extended LD pattern present in the MHC region, it is not clear whether the markers tested in this study confer risk of psoriasis dependently or independently of HLA-C*06:02. Our LD analysis and association with disease or with PASI score indicate that the possibility of a HLA-C*06:02-independent effect for rs887466 and rs1062470, respectively, is much higher than for rs2894207 and rs10484554; however, confirmation of this requires additional studies in this and other populations.

## Electronic supplementary material

Below is the link to the electronic supplementary material.

Supplementary material 1 (PDF 81 KB)
