# Supplementary material for: HLA-C*06:02-independent, gender-related association of PSORS1C3 and PSORS1C1/CDSN single-nucleotide polymorphisms with risk and severity of psoriasis
Source: Mol Genet Genomics. 2018 Mar 27;293(4):957–66. doi: 10.1007/s00438-018-1435-4 (PMC6061044; doi:10.1007/s00438-018-1435-4)
Supplement: Supplementary file 1 — Supplementary material 1 (PDF 81 KB) [file 438_2018_1435_MOESM1_ESM.pdf]

Supplementary Table S1. Characteristics of the SNPs examined in this study and result of test for departure from Hardy-Weinberg equilibrium

Chr.	Gene /Locus	SNP	Position on chromosome	Assay ID	HWE Cases p-value*	HWE Controls p-value*
6	*PSORS1C1/CDSN*	rs1062470	31116658	C__2438414_20	0.01	0.28
6	*PSORS1C3*	rs887466	31175734	C__8941351_1_	0.01	0.56
6	*LOC105375015*	rs2894207	31295974	C__16090306_20	0.0005	0.30
6	*LOC105375015*	rs10484554	31306778	C__29612773_20	0.006	1.0

Chr. - chromosome; *departure from Hardy Weinberg equilibrium was if $p < 0.05$

Supplementary Table S2. Distribution of rs887466 genotypes in men and women

	rs887466	Cases	Controls	p	OR (95%CI)	P ($\chi^2_{df=2}$)
Men	GG	110 (40.6)	70 (26.4)	-	1*	p=0.0002
	GA	134 (49.4)	144 (54.3)	0.007	0.59 (0.40-0.87)	
	AA	27 (10.0)	51 (19.2)	0.0001	0.34 (0.19-0.59)	
Women	GG	60 (31.6)	66 (34.9)	-	1*	p=0.10
	GA	106 (55.8)	87 (46.0)	0.20	1.34 (0.85-2.10)	
	AA	24 (12.6)	36 (19.1)	0.34	0.73 (0.39-1.37)	

*- baseline group

Supplementary Table S3. Correlation of four *PSORS1* SNPs with PASI score value in psoriatic females (N=135)

SNP	BETA	SE	R^2	T	P
rs1062470	-0.033	0.897	1.038e-005	-0.037	0.970
rs887466	-0.070	0.976	3.929e-005	-0.072	0.942
rs2894207	0.909	0.947	0.0068	0.960	0.338
rs10484554	0.808	0.935	0.0055	0.863	0.389
HLA-C*06:02	0.931	1.041	0.0059	0.894	0.372

BETA – regression coefficient, SE – standard error, R^2 – regression r-sqared, T – Wald test (based on t-distribution), p - Wald test asymptotic p-value

Supplementary Table S4. Linkage disequilibrium pattern for controls (N=454). The plot shows r^2 value as a pairwise measure of LD. Dark grey shading indicates strong LD.

	rs1062470	rs887466	HLA-C*06:02	rs2894207	rs10484554
rs1062470	-	0.01	0.26	0.07	0.05
rs887466		-	0.1	0.01	0.001
*HLA-C*06:02*			-	0.33	0.47
rs2894207				-	0.72
rs10484554					-

Supplementary Table S5. Linkage disequilibrium pattern for patients (N=461). The plot shows r^2 value as a pairwise measure of LD. Dark grey shading indicates strong LD.

	rs1062470	rs887466	HLA-C*06:02	rs2894207	rs10484554
rs1062470	-	0.01	0.44	0.15	0.13
rs887466		-	0.21	0.003	0.02
*HLA-C*06:02*			-	0.47	0.57
rs2894207				-	0.80
rs10484554					-
